# Tofacitinib Use in a Patient With Rheumatoid Arthritis and Polycythemia Vera: A Case Report

**DOI:** 10.1002/ccr3.71730

**Published:** 2026-01-02

**Authors:** Milan Bogojevic, Rifat Medjedovic, Milica Markovic, Nikola Bakic, Dragana Pravilovic Lutovac

**Affiliations:** ^1^ Clinical Center of Montenegro Department of Rheumatology Podgorica Montenegro; ^2^ Clinical Center of Montenegro Hematology Center Podgorica Montenegro

**Keywords:** hydroxyurea, jak inhibitors, polycythemia vera, rheumatoid arthritis, tofacitinib

## Abstract

Rheumatoid arthritis (RA) is a chronic autoimmune disease characterized by synovial inflammation and joint destruction, whereas polycythemia vera (PV) is a myeloproliferative neoplasm driven by the Janus kinase (JAK)2 V617F mutation, resulting in erythrocytosis and increased thromboembolic risk. Although JAK inhibitors are established in rheumatology, most selectively inhibit JAK1 and JAK3, sparing JAK2 activity, which is central to PV pathogenesis. We describe a 61‐year‐old woman with seropositive RA and JAK2 V617F‐positive PV. Treatment for polycythemia vera included hydroxyurea. Methotrexate and sulfasalazine were prescribed for rheumatoid arthritis but were withdrawn due to intolerance, and therapy was switched to tofacitinib 5 mg twice daily. Within 3 months, she achieved clinical and ultrasonographic remission of RA, with improvement of inflammatory markers. Hematologic parameters remained stable throughout follow‐up. After 6 months, a mild improvement allowed reduction in hydroxyurea dosing, although further tapering resulted in recurrence of erythrocytosis. After 12 months of therapy, RA remission persisted without adverse events or cytopenias. Tofacitinib demonstrated rheumatologic efficacy but minimal effect on PV‐related hematologic activity.

## Key Clinical Message

- Tofacitinib effectively induced remission of rheumatoid arthritis in a patient with concomitant polycythemia vera, demonstrating strong rheumatologic efficacy while maintaining hematologic safety.
- Its limited impact on erythrocytosis underscores the need for JAK2‐directed therapies when treating patients with overlapping autoimmune and myeloproliferative diseases.

## 1 Introduction

The Janus kinase (JAK)/signal transducer and activator of transcription (STAT) pathway plays a central role in both autoimmune and myeloproliferative disorders. In rheumatoid arthritis (RA), selective JAK inhibitors—such as tofacitinib, baricitinib, and upadacitinib—suppress cytokine‐mediated inflammation [1]. In contrast, PV is driven by constitutive activation of JAK2 signaling due to the V617F mutation, leading to uncontrolled erythropoiesis [2]. Previous reports have demonstrated that ruxolitinib, a selective JAK1/JAK2 inhibitor, can improve both PV and inflammatory bowel disease activity [3]. Experimental data also suggest that low‐dose methotrexate may inhibit JAK/STAT signaling and ameliorate myeloproliferative features in PV models [4, 5]. Recent comprehensive reviews have highlighted that JAK inhibitors differ substantially in their selectivity profiles, with first‐generation agents such as tofacitinib showing predominant JAK1/JAK3 inhibition and only modest activity against JAK2. This selectivity has important clinical implications, as JAK2 plays a central role in hematopoiesis and mediates signaling downstream of erythropoietin and thrombopoietin receptors, making JAK2‐sparing inhibitors less effective for myeloproliferative conditions such as PV. These mechanistic insights underscore why tofacitinib, despite its broad immunomodulatory effects, would be expected to control autoimmune inflammation but exert limited impact on JAK2‐driven disorders, in contrast to JAK1/JAK2 inhibitors such as ruxolitinib [6]. However, there are no published reports on the use of rheumatologic JAK inhibitors, such as tofacitinib, in patients with concomitant RA and PV.

## 2 Case Presentation

### 2.1 Case History / Examination

A 61‐year‐old woman (born 1957) was diagnosed in February 2018 with seropositive rheumatoid arthritis based on chronic symmetric polyarthritis and positive autoantibodies. At presentation in 2018, laboratory testing showed erythrocytosis and leukocytosis with the following values: erythrocytes 6.99 × 10^6^/μL, hemoglobin 155 g/L, hematocrit 0.51, leukocytes 30.5 × 10^9^/L with 89% neutrophils, and platelets 595 × 10^9^/L. Inflammatory and immunologic markers were elevated (rheumatoid factor (RF) 126 IU/mL, anti‐cyclic citrullinated peptide (CCP) > 300 U/mL). Hydroxychloroquine and corticosteroids were started, while disease‐modifying antirheumatic drugs (DMARDs) were postponed pending hematologic evaluation. In August 2018, bone marrow biopsy demonstrated a myeloproliferative neoplasm consistent with polycythemia vera, with reticulin fibrosis grade MF‐1/2, and negative BCR‐ABL (Philadelphia chromosome). The patient was initiated on low‐dose aspirin and cytoreductive therapy with hydroxyurea 500 mg 2 × 1 and 1 × 1 on alternating days. Two therapeutic phlebotomies were performed between 2018 and 2021. Splenomegaly (15 cm) was noted on ultrasound. The patient was lost to rheumatology follow‐up from August 2018 to June 2021, when methotrexate in dose 17.5 mg once a week was introduced but later discontinued in October 2023 due to gastrointestinal intolerance. Sulfasalazine was subsequently prescribed October 2023 but caused a diffuse skin rash. Whole time between 2018 and 2021, the patient was treated continuously with hydroxyurea 500 mg 2 × 1 and 1 × 1 on alternating days and low‐dose aspirin. Two therapeutic phlebotomies were performed during this period due to elevated hematocrit values. In April 2024, she was hospitalized for evaluation for biologic therapy, with disease activity score (DAS)28 score of 5.27, indicating high disease activity. Because she did not meet hematologic criteria for ruxolitinib initiation, tofacitinib 5 mg twice‐daily was started in June 2024 while continuing hydroxyurea 500 mg (2 × 1 and 1 × 1 on alternating days).

### 2.2 Methods (Differential Diagnosis, Investigations, and Treatment)

**FIGURE 1:**
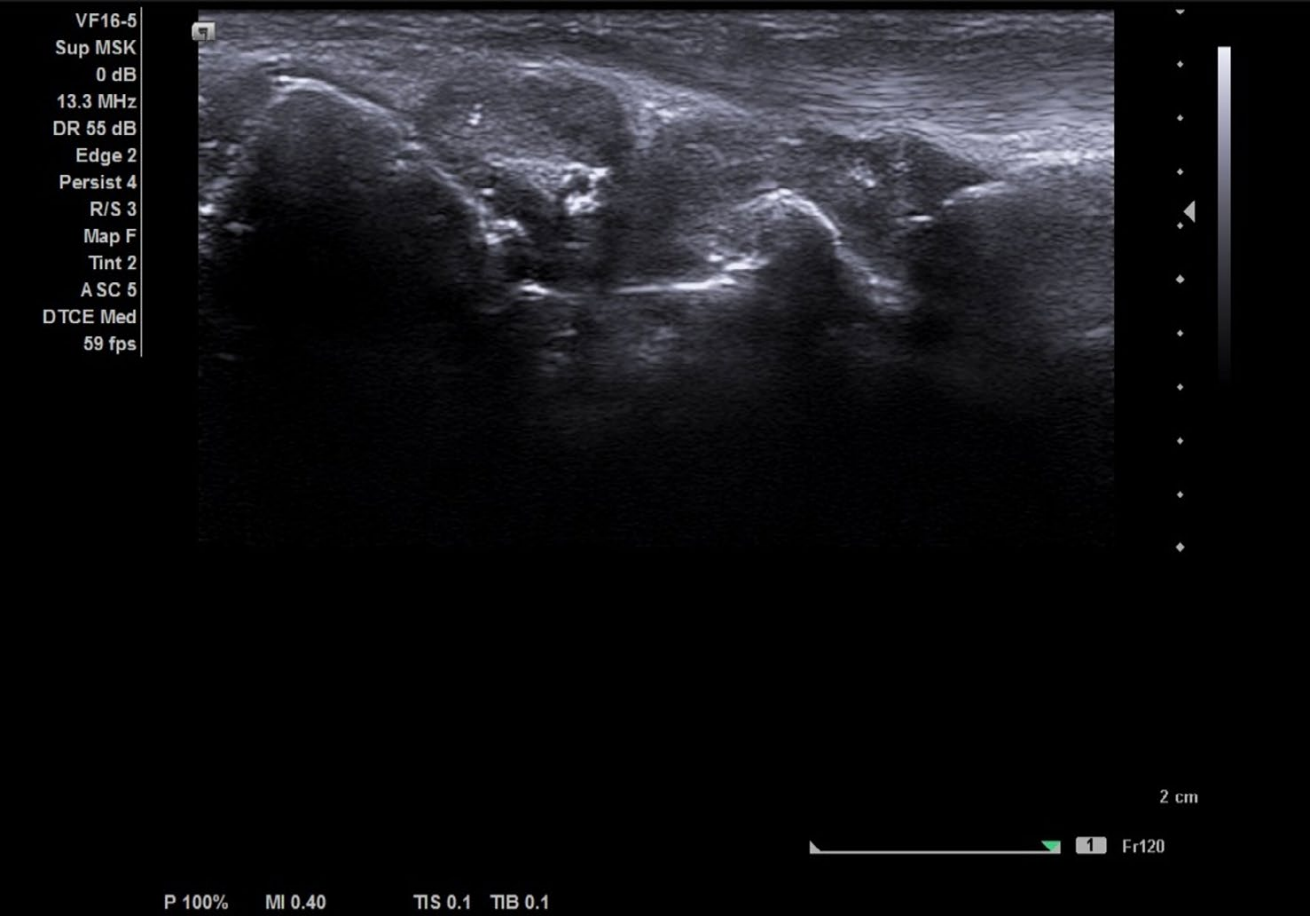
Ultrasound picture of radiocarpal joint on gray scale. Longitudinal ultrasound image of the radiocarpal joint showing the distal radius (left), lunate (center), and capitate (right) as hyperechoic cortical lines. A marked anechoic synovial effusion with synovial hypertrophy (grade III synovitis) is visible over the joint surfaces.

Differential diagnoses at RA onset included undifferentiated connective tissue disease and reactive myeloproliferation secondary to inflammation, but persistent erythrocytosis and the JAK2 V617F mutation with polymerase chain reaction (PCR) confirmed PV. Laboratory monitoring included complete blood count (CBC), C‐reactive protein, erythrocyte sedimentation rate (ESR), and liver and renal function tests every 6–8 weeks. Disease activity was assessed by DAS28‐ESR and musculoskeletal ultrasound (Figure 1). A JAK inhibitor was selected over biological DMARDs because the patient lives in a remote area and was poorly motivated to attend regular visits, making subcutaneous or intravenous therapies impractical and contributing to her historically irregular follow‐up. Tofacitinib was selected due to its pan‐JAK activity and potential partial JAK2 inhibition, and Baricitinib was not available for clinical use in our country at the time [1]. Hydroxyurea was maintained at the lowest effective dose. No anticoagulant complications were reported during therapy.

### 2.3 Conclusions and Results (Outcome and Follow‐Up)

Within 3 months of starting tofacitinib, the patient achieved clinical and ultrasonographic remission of RA, with normalization of inflammatory markers and resolution of joint pain and stiffness that was sustained during follow‐up. Hematologic evaluation after 6 months showed stable erythrocytosis and thrombocytosis, with a mild improvement that allowed reduction in hydroxyurea to 500 mg once‐daily. Dose adjustments of hydroxyurea corresponded closely to hematologic trends. A reduction in dose was attempted after three and 6 months of tofacitinib therapy when hemoglobin and hematocrit improved; however, recurrence of erythrocytosis (Hgb 157 g/L, Hct 0.486) required re‐escalation to 500 mg daily (Table 1). Further tapering was not possible, as hematocrit remained above target levels. No adverse events or cytopenias were observed. Thus, while tofacitinib effectively controlled autoimmune inflammation, its impact on PV was limited, suggesting insufficient inhibition of JAK2 signaling at therapeutic doses used in rheumatology.

**TABLE 1:** Hematologic and inflammatory parameters over time.

Parameter	2018 presentation	June 2024 (pre‐tofacitinib)	3 months after tofacitinib	6 months after tofacitinib	12 months after tofacitinib
RBC (×10^6^/μL)	6.99	5.1	3.9	4.5	4.8
Hgb (g/L)	155	134	127	140	140
Hct	0.51	0.46	0.396	0.401	0.432
WBC (×10^9^/L)	30.5	12.3	11.3	17.2	14.2
Neutrophils (×10^9^/L)	25.4	9.8	9.7	15.3	11.3
Platelets (×10^9^/L)	595	360	259	225	250
ESR (mm/h)	28	31	14	10	8
CRP (mg/L)	6.2	7.5	3.5	0.5	0.5

## 3 Discussion

This case illustrates the differential effects of JAK inhibition across diseases driven by distinct JAK isoforms. Although tofacitinib is classified as a pan‐JAK inhibitor, it preferentially targets JAK1 and JAK3, with weaker inhibition of JAK2, which is essential in hematopoiesis [1, 2]. The discussion of methotrexate's indirect effects on JAK–STAT signaling has been abbreviated, as these mechanisms are secondary to the central focus on tofacitinib's pharmacologic profile [4, 5]. In contrast, ruxolitinib, a selective JAK1/JAK2 inhibitor, has demonstrated efficacy in controlling both PV and inflammatory disorders such as ulcerative colitis [3]. This pharmacologic distinction is clinically relevant, as large trials of the JAK1/JAK2 inhibitor ruxolitinib have demonstrated robust hematocrit control, spleen volume reduction, and durable hematologic remission in patients with hydroxyurea‐resistant PV, confirming that effective management of PV requires meaningful JAK2 blockade [7]. The limited hematologic response in our patient supports the notion that JAK2 inhibition is necessary for meaningful PV control. The translational relevance of JAK inhibitor selectivity is particularly evident in this case. Tofacitinib's preferential inhibition of JAK1 and JAK3, with only modest activity against JAK2 as highlighted by Schwartz et al. limits its impact on erythropoiesis while maintaining potent anti‐inflammatory effects. This JAK2‐sparing profile may reduce the risk of cytopenias in PV patients requiring RA therapy, offering a potentially safer immunomodulatory option in individuals with underlying myeloproliferative disease [6]. Advances in exosome biology have shown that extracellular vesicles actively shape cytokine and JAK–STAT signaling in hematologic malignancies, emphasizing that immune dysregulation in PV extends beyond clonal proliferation alone [8]. Nevertheless, this case highlights the safety of tofacitinib in patients with MPN, without hematologic deterioration, and suggests potential for multidisciplinary evaluation in patients with overlapping autoimmune and myeloproliferative disorders. Future development of JAK2‐selective or dual‐JAK inhibitors may help bridge the therapeutic gap for patients with concurrent RA and PV, offering more comprehensive control of both inflammatory and myeloproliferative activity.

## 4 Conclusion

Tofacitinib induced complete remission of rheumatoid arthritis in a patient with concomitant polycythemia vera, but its effect on hematologic parameters was minimal. Tofacitinib was effective and well‐tolerated in a patient with RA and PV, underscoring its safety but limited JAK2 inhibition. JAK2‐targeted agents may provide broader benefit in such overlap syndromes. Further studies are needed to explore the safety and efficacy of JAK inhibitors across overlapping autoimmune and hematologic diseases.

## Author Contributions

**Milan Bogojevic:** conceptualization, data curation, investigation, validation, writing – original draft, writing – review and editing. **Rifat Medjedovic:** data curation, methodology, resources, writing – original draft, writing – review and editing. **Milica Markovic:** data curation, investigation, methodology, resources, writing – original draft, writing – review and editing. **Nikola Bakic:** conceptualization, data curation, investigation, methodology, writing – review and editing. **Dragana Pravilovic Lutovac:** conceptualization, investigation, methodology, project administration, resources, visualization, writing – review and editing.

## Funding

The authors have nothing to report.

## Ethics Statement

The authors have nothing to report.

## Consent

Written informed consent was obtained from the patient to publish this report in accordance with the journal's patient consent policy.

## Conflicts of Interest

The authors declare no conflicts of interest.

## Data Availability

Data sharing not applicable to this article as no datasets were generated or analyzed during this study.

## References

[1] S. Dhillon , “Tofacitinib: A Review in Rheumatoid Arthritis,” Drugs 77, no. 18 (2017): 1987–2001. 29139090 10.1007/s40265-017-0835-9

[2] E. J. Baxter , L. M. Scott , P. J. Campbell , et al., “Acquired Mutation of the Tyrosine Kinase JAK2 in Human Myeloproliferative Disorders,” Lancet 365, no. 9464 (2005): 1054–1061. 15781101 10.1016/S0140-6736(05)71142-9

[3] E. C. Swei , C. M. Fox , D. W. Bowles , M. N. Rizeq , and J. C. Onyiah , “Use of Ruxolitinib for the Simultaneous Treatment of Severe Refractory Ulcerative Colitis and Polycythemia Vera,” ACG Case Reports Journal 9 (2022): e00741. 35018293 10.14309/crj.0000000000000741 PMC8740881

[4] K. Chinnaiya , M. A. Lawson , S. Thomas , et al., “Low‐Dose Methotrexate in Myeloproliferative Neoplasm Models,” Haematologica 102, no. 6 (2017): e336–e339. 28550185 10.3324/haematol.2017.165738 PMC5685234

[5] S. Francis , S. Thomas , R. Luben , et al., “Low‐Dose Methotrexate: Potential Clinical Impact on Haematological and Constitutional Symptoms in Myeloproliferative Neoplasms,” British Journal of Haematology 187, no. 5 (2019): 655–659. 10.1111/bjh.16193 31525819

[6] D. M. Schwartz , Y. Kanno , A. Villarino , M. Ward , M. Gadina , and J. J. O'Shea , “JAK Inhibition as a Therapeutic Strategy for Immune and Inflammatory Diseases,” Nature Reviews. Drug Discovery 16, no. 12 (2017): 843–862. 29104284 10.1038/nrd.2017.201

[7] S. Verstovsek , A. M. Vannucchi , M. Griesshammer , et al., “Ruxolitinib Versus Best Available Therapy in Patients With Polycythemia Vera: 80‐Week Follow‐Up From the RESPONSE Trial,” Haematologica 101 (2016): 821–829. 27102499 10.3324/haematol.2016.143644 PMC5004461

[8] K. Ghaffari , A. Moradi‐Hasanabad , A. Sobhani‐Nasab , J. Javaheri , and A. Ghasemi , “Application of Cell‐Derived Exosomes in the Hematological Malignancies Therapy,” Frontiers in Pharmacology 14 (2023): 1263834. 37745073 10.3389/fphar.2023.1263834 PMC10515215

